# Effect of Previous Frozen Storage, Canning Process and Packing Medium on the Fatty Acid Composition of Canned Mackerel

**DOI:** 10.3390/md20110666

**Published:** 2022-10-25

**Authors:** Ricardo Prego, Marcos Trigo, Beatriz Martínez, Santiago P. Aubourg

**Affiliations:** 1 Department of Oceanography, Marine Research Institute (CSIC), 36208 Vigo, Spain; 2 Department of Food Technology, Marine Research Institute (CSIC), c/Eduardo Cabello, 6, 36208 Vigo, Spain; 3 Department of Food Technologies, CIFP Coroso, Avda. da Coruña, 174, 15960 Ribeira, Spain

**Keywords:** Atlantic mackerel, frozen storage, packing medium, canning, fatty acids, polyunsaturated, ω3/ω6 ratio, EPA, DHA

## Abstract

This study addressed the fatty acid (FA) composition of canned Atlantic mackerel (*Scomber scombrus*). In it, the effect of prior frozen storage (6 months at −18 °C), different packing media (water, brine, and sunflower, refined and extra virgin olive oils), and canning procedure was investigated. As a result, the canning procedure led to a decrease (*p* < 0.05) in saturated FA (STFA) levels, an increase (*p* < 0.05) in polyunsaturated FA (PUFA) and total ω3 FA values, and higher PUFA/STFA and ω3/ω6 ratio values. Concerning the packing medium effect, the great presence of C18:2ω6 in sunflower oil led to high PUFA and PUFA/STFA values and low ω3/ω6 ratios when compared to other packing media. However, the high presence of C18:1ω9 in both olive oils tested did not lead to remarkable increases (*p* > 0.05) of this FA presence. Additionally, the presence of total ω3 FAs, C20:5ω3 and C22:6ω3 did not provide differences in canned fish muscle as a result of using different packing media. In all canned samples, ω3/ω6 values were included in the 8.2–10.8 range. Prior frozen storage did not have a substantial effect (*p* > 0.05) on the FA group (STFA, monounsaturated FA, PUFA, total ω3 FA) and FA ratio (PUFA/STFA and ω3/ω6) values.

## 1. Introduction

Seafood consumption has increased in recent decades, providing a high content of important constituents for the human diet, such as nutritional and digestive proteins, lipid-soluble vitamins (namely, A and D), microelements (*I*, *F*, *Ca*, *Cu*, *Zn*, *Fe* and others) and highly unsaturated fatty acids [1]. In this context, marine lipids are now the subject of a great deal of attention due to their high content of polyunsaturated fatty acids (PUFAs); among PUFA compounds, special attention has been paid to ω3 fatty acids (FAs), i.e., eicosapentaenoic (C20:5ω3, EPA) and docosahexaenoic (C22:6ω3, DHA) acids), on the basis of their positive role in preventing certain human diseases [2,3]. In this context, it is now recognised that most Western countries do not consume adequate levels of FAs belonging to the ω3 series, with great attention on the ω3/ω6 ratio of foods included in the human diet [4,5]. In order to prevent relevant health disorders, ω3/ω6 ratios varying from 1/4 to 1/1 have been recommended depending on the disease under consideration [6].

However, seafood constitutes highly perishable products whose quality rapidly declines post-mortem as a result of processing and storage. Marine species, due to their chemical composition, pH close to neutrality and high-water content, are an excellent media for microbial growth and enzymatic reactions [7,8]. Such food deteriorates after death due to the development of different damage pathways [9]. Since marine lipid composition includes a high content of PUFA compounds, the development of lipid oxidation during processing is likely to occur, especially if thermal treatment and a fatty fish species are concerned. Consequently, important losses of unsaturated FAs are likely to be produced and lead to detrimental effects on nutritional and sensory values [10,11].

Among traditional technologies, canning represents one of the most important means of marine species preservation [12,13]. Recent FAO statistics (2010–2019) have reported that about 9.5–11.5% (ca. 16.3–17.5 million tons per year) of total world fishery production would correspond to marine species canning [14]. In this process, seafood is introduced in sealed hermetic containers in the company of different kinds of packing media (oil, brine, pickle, etc.) and typically encompasses a sterilisation procedure, in which the canned fish tissue is heated in a temperature range of 110–130 °C for a period of 25–120 min. The extensive heat treatment involved alters the nature of the raw material, so that a product with different characteristics is formed. As a result of heat treatment, both enzymes and bacteria should be permanently inactivated [15,16]. Thus, a wide range of fish and invertebrate species produce excellent canned products, supporting an important role in human nutrition. Unfortunately, most species destined for canning are caught in large quantities and canneries have to store the raw material before it is processed. Consequently, most of the problems with canned fish acceptance can be related to the quality of the raw material, which continuously changes during storage prior to processing [9,17]. Previous research accounts for studies focused on chemical changes and quality loss in canned seafood as a result of the thermal treatment involved, packing medium employed and prior storage conditions applied. Such studies have addressed biogenic amine formation [18], changes in physical properties [19,20], amino acid and protein profile modifications [21,22], lipid damage [23] and the presence of metals [24]. Furthermore, the preservative effect of an antioxidant addition in the packing medium has been proved [25,26]. Concerning the FA profile changes, most research has addressed the effect of packing media [17,27,28]; on the contrary, only a few studies have addressed the effect of thermal treatment (sterilisation) during canning, and the conditions of prior storage to canning [29,30].

In the present work, the FA composition of canned Atlantic mackerel (*Scomber scombrus*) was addressed. The effect of prior frozen storage (6 months at −18 °C), different packing media (water, brine, and sunflower, refined and extra virgin olive oils), and canning procedure was investigated. This study focused on FA groups (saturated FAs, STFAs, monounsaturated FAs, MUFAs, PUFAs, total ω3 FAs) and FA ratios (PUFA/STFA and ω3/ω6) in initial and canned mackerel muscle. According to their high nutritional significance, the evolution of the EPA and DHA contents was analysed. Changes in the moisture and water values of mackerel muscle were also evaluated.

## 2. Results and Discussion

### 2.1. Moisture and Lipid Content of Initial and Canned Mackerel Muscle

Moisture and lipid values (Table 1) corresponding to initial fish agree with those reported for mackerel and other fatty fish species [31,32,33]. A comparison of initial mackerel tissue and canned mackerel without having undergone a prior frozen storage period revealed a marked decrease in average moisture value in all kinds of canned samples (Table 1); differences were found to be significant (*p* < 0.05) in all cases, except for the brine-packed batch. A general decrease in average moisture value in canned fish was detected as a result of the prior 6-month frozen storage period (termed “6-canned”); this decrease was found to be significant (*p* < 0.05) in all batches except for canned samples packed with refined olive oil. For both 0-canned and 6-canned samples, no significant differences (*p* > 0.05) were detected as a result of the packing medium employed. The results showed that prior frozen storage and canning procedure led to a decrease in moisture content in canned fish. No effect of the packing medium was found on the observed decrease in moisture content.

The evolution of the lipid content of canned fish indicated great differences according to the nature of the packing medium employed (Table 1). Thus, a comparison between initial and 0-canned fish revealed a substantial lipid increase (*p* < 0.05) with canning when aqueous media (i.e., water or brine) were employed. On the contrary, lower average values were detected in canned fish if applying an oily packing medium; differences were found to be significant (*p* < 0.05) if sunflower oil was used. A general increase in average lipid content in canned fish was detected as a result of the prior holding time, differences being significant (*p* < 0.05) for canned fish including aqueous packing media. For both 0-canned and 6-canned samples, fish corresponding to oil-packed samples showed lower (*p* < 0.05) lipid values than their counterparts from aqueous media. The results showed that the polar nature of packing medium has a marked effect on the lipid content of canned mackerel tissue.

In the present study, moisture and lipid contents in canned fish can be influenced by different damage mechanisms. On the one hand, protein denaturation during frozen storage, and especially during the canning procedure, can lead to a decrease in the water-holding capacity of fish muscle, so that a water release into the packing medium would be produced [9,34]. As a result of this moisture content decrease, lipids and other constituents would increase their relative content in canned muscle. On the other hand, the presence of an oily medium as a coating would partially extract the lipid fraction of the muscle, so that a lipid content decrease would be produced. This decreasing effect would not take place in fish canned with aqueous packing media and would explain the higher lipid content found in the current study in fish canned with aqueous packing when compared to their counterparts corresponding to oily packing.

Another aspect to be taken into account is the fact that protein has become degraded as a result of the canning procedure and may diffuse out of the muscle into the surrounding packing medium; this effect would be especially important in an aqueous-packing medium. This protein loss would lead to a relative increase in the lipid content in the muscle and could partly explain the lipid content increase detected in canned fish corresponding to both aqueous-packing media. Protein degradation loss from the canned muscle could be increased in fish previously subjected to frozen storage [7,34]. This fact would explain the general higher average lipid values observed in 6-canned fish when compared to their counterparts from the 0-canned batch.

In most cases, previous research has shown a moisture content decrease in fish muscle as a result of the canning procedure [35,36]. However, different and contradictory results have been shown for the lipid content depending on the use of an aqueous or an oily packing medium. Additionally, the content of both constituents has shown a marked dependency on the extraction degree of the coating medium before canned muscle analysis. Thus, a comparative packing study was carried out on Little Tunny (*Euthynnus alletteratus*) [37]; as a result, a decrease in moisture and lipid contents was detected in brine-canned fish, while fish canned in olive oil led to lower moisture values and no differences in the lipid content. A great absorption of packing oil into the fish muscle was reported during soybean-packed tuna (*Thunnus alalunga*) [21] and coconut-, sunflower- and groundnut-packed yellowfin tuna (*Thunnus albacares*) [38]; additionally, moisture loss in canned fish was detected in such studies. Fish packing with an aqueous medium led to a higher moisture content and a lower lipid value than fish that was packed by using an oily coating medium [28,39]. When applying a sunflower oil-packing medium, a moisture value decrease and a lipid content increase were detected in canned Coho salmon (*Oncorhynchus kisutch*) as a result of canning [40]. Recently, a substantial decrease in moisture content and increase in lipid value was observed in water-packed Chub mackerel (*Scomber colias*) by Malga et al. [35] as a result of the canning procedure.

Concerning the effect of prior fish-holding time, no effect of prior frozen storage time (0–15-month period at −18 °C) of sardine (*Sardina pilchardus*) was detected on the water and lipid contents of the corresponding canned product [41]. However, an increase in lipid content in canned sardine (*S. pilchardus*) was proved by increasing the prior holding time in ice [30]; on the contrary, no effect on moisture level was reported.

### 2.2. FA Composition of Initial Packing Oils and Initial Mackerel Muscle

The composition of initial oils employed as packing media is shown in Table 2. A very different FA composition was detected in sunflower oil when compared to both olive oils. The two major FAs in sunflower oil were C18:2ω6 and C18:1ω9 (ca. 55% and 31%, respectively); additionally, two relatively abundant FAs were C16:0 and C18:0. For both olive oils, the most abundant FA was C18:1ω9 (ca. 74%), followed by C16:0 (ca. 12–13%). Other relatively abundant FAs were C18:2ω6, C18:0 and C18:1ω7.

The values for FA groups and the ratios of initial packing oils are presented in Table 3. According to individual FA composition, the following decreasing sequence was detected for FA groups in sunflower oil: PUFA > MUFA > STFA. A different decreasing sequence was observed for both olive oils: MUFA > STFA > PUFA. Higher (*p* < 0.05) PUFA/STFA ratios were detected in sunflower oil than in both olive oils. Furthermore, ω3/ω6 ratio values and even total ω3 levels were found to be negligible in all initial oils. Total ω6 content was especially high in sunflower oil according to the great content on C18:2ω6.

Concerning the initial mackerel muscle, a very different FA composition was observed when compared to any of the packing oils used (Table 2). The most abundant FAs were C16:0, C18:1ω9 and C22:6ω3, other abundant FAs being C20:5ω3, C18:0, C16:ω7, C18:1ω7 and C14:0. Such FA composition agrees with previous research carried out on wild fatty fish species [31,33,36]. Each of the FA groups, STFA, MUFA and PUFA, revealed values included in the 30–36% range, with PUFA/STFA and ω3/ω6 ratios reaching valuable levels round 0.9 and 9.0, respectively. Additionally, the total ω3 level was around 27%, EPA and DHA being the major components of this FA group (ca. 8.5% and 17.0%, respectively).

In order to better focus on possible changes in the FA composition of mackerel muscle, a discussion of the FA results is addressed to the FA groups (STFA, MUFA, PUFA, and total ω3) and FA ratios (PUFA/STFA and ω3/ω6) in the next sections. Additionally, and based on the great significance of EPA and DHA [2,3], changes in their contents are also discussed individually.

### 2.3. Effect of Canning Procedure on the FA Composition of Canned Mackerel Muscle

**Figure 1:**
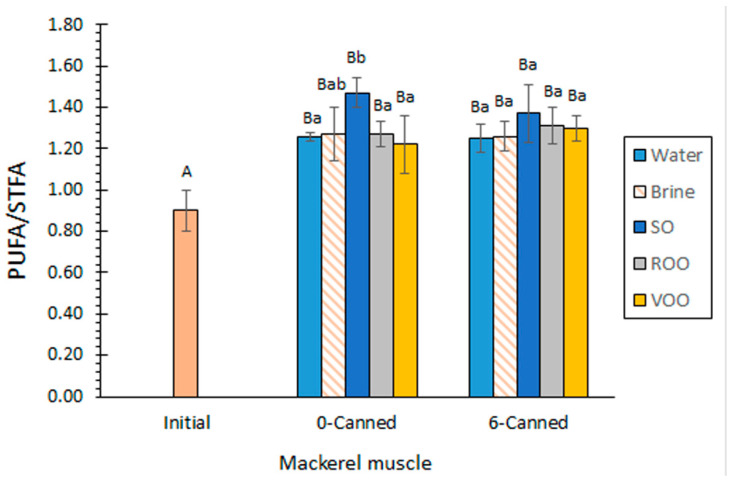
Determination of PUFA/STFA ratio in initial and canned mackerel muscle including different packing media. Average values of five replicates (*n* = 5); standard deviations are indicated by bars. For each packing medium, average values followed by different capital letters (A,B) denote significant differences (*p* < 0.05) as a result of canning procedure and prior frozen storage. For each prior holding condition, average values followed by different lowercase letters (a,b) denote significant differences (*p* < 0.05) as a result of packing medium. Abbreviations: SO (sunflower oil), ROO (refined olive oil), VOO (extra virgin olive oil), PUFA (polyunsaturated fatty acid), STFA (saturated fatty acid), 0-canned (canned fish without prior frozen storage) and 6-canned (canned fish with prior 6-month frozen storage).

The composition of the initial fish and the canned muscle without prior frozen storage (i.e., 0-canned fish) revealed substantial changes in the FA groups as a result of the canning procedure in all kinds of packed fish (Table 4). The different types of canned mackerel products showed lower (*p* < 0.05) STFA levels than the initial fish. On the contrary, a substantial increase (*p* < 0.05) in PUFA content was detected in all canned batches. Concerning the MUFA presence, no effect (*p* > 0.05) was implied for this FA group as a result of the canning procedure, except for the sunflower oil batch. According to this distribution of FA groups, all kinds of 0-canned fish showed an increase (*p* < 0.05) in PUFA/STFA ratio (Figure 1), all values being included in the 1.22–1.47 range.

**Figure 2:**
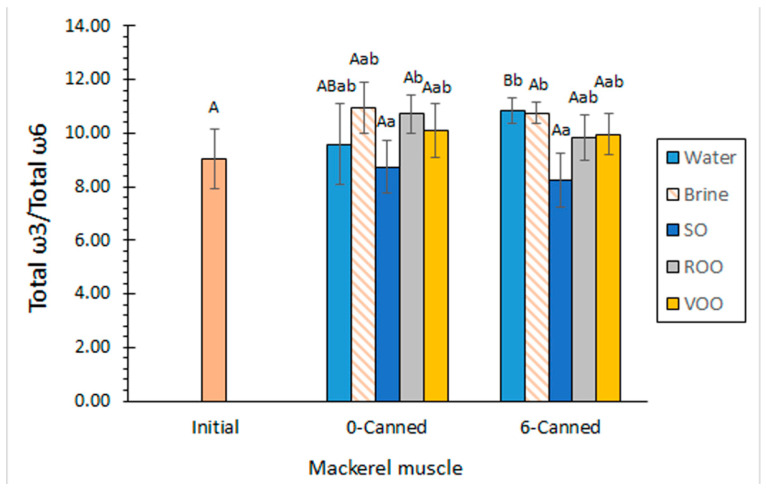
Determination of ω3/ω6 ratio in initial and canned mackerel muscle including different packing media. Average values of five replicates (*n* = 5); standard deviations are indicated by bars. For each packing medium, average values followed by different capital letters (A,B) denote significant differences (*p* < 0.05) as a result of canning procedure and prior frozen storage. For each prior holding condition, average values followed by different lowercase letters (a,b) denote significant differences (*p* < 0.05) as a result of packing medium. Abbreviations: SO (sunflower oil), ROO (refined olive oil), VOO (extra virgin olive oil), 0-canned (canned fish without prior frozen storage) and 6-canned (canned fish with prior 6-month frozen storage).

Related to the total ω3 FA content (Table 5), a comparison of average values revealed a general increase in all kinds of samples as a result of the canning procedure. This increase was found to be significant (*p* < 0.05) in all cases except for canned fish including brine as a packing medium. The analysis of the ω3/ω6 ratio showed an average increase with the thermal process in most cases (Figure 2), fish canned in sunflower oil being the only exception. All canned values were included in the 8.7–11.0 range, which according to nutritional recommendations can be considered as highly valuable [6].

According to the results obtained for the total ω3 FAs, the analysis of the EPA and DHA presence (Table 5) in fish muscle revealed a substantial increase in average values after the canning procedure. For EPA content, differences were found to be significant (*p* < 0.05) in canned fish including an aqueous filling medium (i.e., water or brine). In the case of DHA level, a significant increase (*p* < 0.05) was found in canned fish including any of the olive-oil filling media tested.

Previous research accounts for studies focused on the effect of the canning procedure on the FA composition of canned fish muscle. No effect of canning was detected in FA composition (individual FAs and FA groups) in brine-canned Little Tunny (*E. alletteratus*) [37]. As a result of canning, a decrease in MUFA content was detected by Naseri and Rezaei [17] in brine-canned sprat (*Clupeonella cultriventris*), while no differences were detected in STFA and PUFA levels; no effect on total ω3 and ω3/ω6 ratio was observed. 

The canning procedure carried out on water-canned Atlantic mackerel (*S. scombrus*) [42] and brine-canned Chub mackerel (*S. colias*) [26,29] did not provide differences in the polyene index (PI) (calculated as the C22:6ω3 + C20:5ω3)/C16:0 FA ratio) of canned muscle. No remarkable effect on FA group contents and PI was detected in water-canned Chub mackerel (*S. colias*) [35]; however, a substantial increase was observed for the ω3/ω6 ratio as a result of canning.

### 2.4. Effect of Packing Medium on the FA Composition of Canned Mackerel Muscle

No effect (*p* > 0.05) of the packing medium could be detected on the STFA content in canned samples corresponding to both 0-canned and 6-canned processing conditions (Table 4). However, some differences could be outlined from the analysis of the MUFA and PUFA presence (Table 4). Canned fish including sunflower oil as a packing medium revealed the lowest average values for MUFA content; for canned fish without prior frozen storage, such differences were found to be significant (*p* < 0.05) by comparison to all other packing media except for brine-canned batch. For canned fish with a prior holding period, differences in MUFA content were only found to be significant (*p* < 0.05) by comparison to brine- and extra virgin olive oil-packed fish. In the case of PUFA presence, the highest average values were detected in canned fish corresponding to sunflower oil-packing medium (Table 4); differences with canned muscle corresponding to other packing media were found to be significant (*p* < 0.05) when compared to water- and refined olive oil-packed (0-canned samples) and to brine-canned (6-canned samples) mackerel muscle.

According to the FA group distribution, the highest average values for the PUFA/STFA ratio were detected in canned fish including sunflower oil as a packing medium (Figure 1). For canned fish without prior storage, differences were found to be significant (*p* < 0.05) by comparison with all canned samples, except for those including brine as a packing medium; on the contrary, differences were not found to be significant (*p* > 0.05) when taking into account samples that were previously stored under frozen conditions.

Concerning the total ω3 FA value, scarce differences were observed as a result of the packing medium employed (Table 5). For canned samples without prior frozen storage, a higher (*p* < 0.05) level was detected in canned samples including sunflower oil as a packing medium when compared to their counterparts corresponding to water and both olive oil media. In the case of canned samples with prior storage, no significant differences (*p* > 0.05) were detected as a result of the packing medium used. 

Average values for the ω3/ω6 ratio revealed the lowest average values in canned samples corresponding to sunflower oil packing (Figure 2); however, no significant differences were observed between sunflower-canned samples and initial ones. For 0-canned fish, differences were found to be significant (*p* < 0.05) by comparison to the refined olive oil-packed batch; in the case of 6-canned samples, differences were found to be significant (*p* < 0.05) by comparison to canned fish including aqueous media.

Concerning the two most valuable ω3 FAs (namely, EPA and DHA; Table 5), no differences (*p* > 0.05) were detected as a result of the packing medium employed in samples that were previously stored under frozen conditions; canned fish corresponding to both olive oil-packed conditions showed the lowest and highest average values for EPA and DHA, respectively. In the case of canned fish without a prior holding period, average EPA levels were higher in samples corresponding to aqueous packing media (i.e., water and brine), differences being significant (*p* < 0.05) by comparison to samples including sunflower oil and extra virgin olive oil. For 0-canned fish, the highest average values for DHA presence were detected in samples including any oily filling media; differences were found to be significant (*p* < 0.05) when compared to water-packed samples.

The present results can be considered the result of two opposite effects. On one side, the absorption of the packing oil into the canned muscle is likely to be produced and lead to an increase in FA content in the muscle of FAs that are present in the packing oil. On the other side, packing oil can act as an extracting system from fish muscle into the packing medium, so that a modification of the FA profile of canned fish can be expected. As expressed in the Material and Methods section, packing media were eliminated in the present study by wrapping the canned fish with filter paper before starting the canned muscle analysis.

The current results show associations between the FA composition of the packing oils with the FA composition of the canned mackerel muscle. Thus, the great presence of C18:2ω6 (Table 2) in sunflower oil led to high PUFA (Table 4) and PUFA/STFA (Figure 1) values and low ω3/ω6 ratios (Figure 2). However, the high presence of C18:1ω9 in both olive oils (Table 2) did not lead to substantial increases in the presence of this FA in the corresponding canned muscle (Table 4). Additionally, the presence of ω3 FAs, measured as total ω3 FA, EPA and DHA contents (Table 5), did not undergo remarkable decreases in fish muscle, even though such FAs are present in very low levels in the initial oils tested in this study. The results showed that a low effect of the packing oil composition was produced on the composition of the canned fish muscle in the current study. This result can be explained on the basis that packing oils were carefully extracted by wrapping the fish muscle with filter paper.

The present results show that no differences (*p* > 0.05) in FA composition were detected by the comparison of canned fish corresponding to both olive oils. It is inferred that the presence of preservative (namely, antioxidants) compounds in extra virgin olive oil [43] did not lead to a greater retention of PUFAs.

Previous research has shown a strong effect of the FA composition of the packing medium employed (i.e., oil packing) on the FA profile of canned fish. Thus, the fat composition of canned tuna (*T. alalunga*) tended to be similar to that of the soya bean oil used for packing [36]; as a result, canned tuna increased in C18:1ω9, C18:2ω6 and C18:3ω3 values, and showed remarkable decreases in C20:4ω6, EPA and DHA levels. A comparative study of two packing conditions (brine and olive oil) of Little Tunny (*E. alletteratus*) was carried out by Aubourg et al. [37]; as a result, a strong presence of the FAs of olive oil was detected in canned tuna packed under such oil medium and leading to higher levels of MUFAs (C18:1ω9, C16:1ω7 and C22:1ω11) and C18:2ω6, but a lower presence of PUFAs (C20:4ω6, EPA and DHA) and total ω3 FAs. Similarly, Ruiz-Roso et al. [27] detected a great influence of olive oil-packing medium in canned sardine (*S. pilchardus*); an increase in MUFA and PUFA presence, but a decrease in STFA, total ω3, EPA and DHA values was detected. Tarley et al. [28] carried out a comparative study of soybean oil and tomato sauce as packing media for sardine (*Sardinella brasiliensis*); as a result, higher levels of C18:2ω6 and C18:3ω3 were observed in soybean oil-canned sardine, while higher levels of EPA and DHA were found in sauce-packed fish. A lower PI was detected in sunflower oil-canned sprat (*C. cultriventris*) when compared to their counterparts packed in brine [17]; a comparison between muscle packed under both packing media revealed higher levels of DHA, EPA, STFAs, MUFAs, total ω3 FAs and ω3/ω6 ratios in brine canned sprat than in their counterparts packed in oil. Recently, Gómez-Limia et al. [44] observed the absorption of the oil used in the canning process in European eel (*Anguilla anguilla*); higher values of C18:1ω9 and MUFAs and decreases in STFA, PUFA and PUFA/STFA values were detected in olive oil-packed eel when compared to their counterparts corresponding to a sunflower oil batch.

### 2.5. Effect of Prior Frozen Storage on the FA Composition of Canned Mackerel Muscle

The effect of the prior holding step can be evaluated by a comparison of 0-canned and 6-canned samples. Thus, the analysis of the STFA, MUFA and PUFA contents did not provide significant differences (*p* > 0.05) as a result of the prior frozen storage (Table 4). A different trend according to the aqueous or oily packing condition employed could not be concluded. According to FA group results, no differences (*p* > 0.05) were detected for the PUFA/STFA value as a result of the prior frozen storage (Figure 1).

No significant differences (*p* > 0.05) were detected for the total ω3 FA values (Table 5), as well as for the ω3/ω6 ratio (Figure 2) as a result of the prior 6-month frozen period. The ω3/ω6 ratio showed a decreasing average value with frozen storage in canned samples corresponding to all packing media, except for those samples including water as a packing medium. According to the results observed for total ω3 FA values, the analysis of the EPA and DHA (Table 5) presence in canned fish did not reveal a significant effect (*p* > 0.05) related to the prior holding period. 

Previous research concerning the effect of prior holding condition on the FA profile of canned fish can be considered scarce. An increased prior frozen storage time (0–15-month period) led to a PI decrease in brine-canned Chub mackerel (*S. colias*) that was explained on the basis of an increased development of the lipid oxidation mechanism [29]. On the contrary, and in agreement with the current study, no substantial effect was detected in the PI of brine-canned sardine (*S. pilchardus*) by increasing the prior holding time on ice [41]. Recently, Reblová et al. [30] detected remarkable decreases in PUFA/STFA and ω3/ω6 ratios in canned sardine (*S. pilchardus*) by increasing the prior chilling time (0–15-day period); however, no effect was observed for the presence of STFA, MUFA and PUFA groups in canned fish.

## 3. Materials and Methods

### 3.1. Initial Fish, Frozen Storage and Chemicals 

Fresh Atlantic mackerel (*S. scombrus*) (110 specimens) (length and weight ranges: 27.5–31.0 cm and 215–255 g, respectively) were obtained at Vigo harbour (North-Western Spain) in November 2020 and transported on ice to the laboratory within 20 min. Ten fish were taken, divided into five groups (two individuals per group), beheaded, eviscerated and filleted. Then, the white muscle was separated, pooled together within each group, minced, analysed independently (*n* = 5) and considered as initial fish.

On the same day, 50 fish were taken and divided into five groups (ten individuals per group). The fish were beheaded, eviscerated, filleted and subjected to the canning procedure (samples without prior frozen storage; 0-canned samples).

The remaining fish (50 specimens) were stored at −40 °C for 48 h and then kept frozen (−18 °C) for 6 months. After this time, individuals were thawed overnight (4 °C) and divided into five groups (ten individuals per group). Fish pieces were beheaded, eviscerated, filleted and subjected to the canning procedure (samples with prior frozen storage; 6-canned samples).

General solvents (chloroform, methanol and toluene) and chemicals (acetyl chloride and NaCl) used were of reagent grade and purchased from Merck (Darmstadt, Germany). Standards and other chemicals were as expressed in the related analytical procedure. Sunflower oil was obtained from Aceites Toledo, S. A. (Toledo, Spain). Olive oils were obtained from Aceites Carbonell S. A. (Alcolea, Córdoba, Spain); both olive oils (Virgen Extra Arbequina and Olive Oil) were prepared from olives obtained in Lleida (Spain).

### 3.2. Canning Process

At each canning time, 45-g portions of mackerel fillets were placed in small flat rectangular cans (105 × 60 × 25 mm; 150 mL). As packing media, water, brine (aq. 2% NaCl solution), sunflower oil, refined olive oil and extra virgin olive oil were employed, respectively. Packing media were added in order to fulfil the corresponding cans. Each can was prepared with a single fish.

The cans were vacuum-sealed and then subjected to the sterilisation process in a horizontal steam retort (115 °C, 45 min; *F*_o_ = 7 min) (CIFP Coroso, Ribeira, A Coruña, Spain). Once the heating time was completed, steam was cut off, and air was used to flush away the remaining steam. The cans were cooled at reduced pressure. Finally, they were stored at room temperature (20 °C) for 3 months. 

A 3-month canned storage was carried out according to common practice employed in canneries. A minimum of 2 months is suggested as necessary by manufacturers in order to optimise fish palatability in commercial canned fish [28]. 

### 3.3. Sampling Procedure 

At each sampling time, the cans were opened, and the liquid part was carefully drained off gravimetrically. Then, the mackerel muscle was separated, and the remaining packing medium was eliminated from the fish muscle by wrapping with filter paper.

The fish white muscle of two cans with the same packing medium was pooled together, minced and employed to carry out the different FA analyses. Cans corresponding to each packing medium were analysed by means of five replicates (*n* = 5).

### 3.4. Assessment of Moisture and Lipid Content in Mackerel Muscle 

The moisture of the fish muscle of initial and canned samples was determined as the weight difference (1–2 g) before and after 4 h at 105 °C, according to the official method 950.46B [45]. The results were calculated as g·kg^−1^ of fish muscle.

The lipids of the fish muscle of initial and canned samples were extracted by the Bligh and Dyer [46] method, which employs a single-phase solubilisation of the lipids using a chloroform–methanol (1:1) mixture. Quantification was carried out according to Herbes and Allen [47]. The results were calculated as g·kg^−1^ of fish muscle.

### 3.5. Analysis of the FA Composition

Lipid extracts of fish muscle (initial and canned samples) and initial oil samples were converted into FA methyl esters (FAME) by using acetyl chloride in methanol and then analysed by gas–liquid chromatography (GLC; Perkin Elmer 8700 chromatograph, Madrid, Spain) [48]. The quantitative response of the equipment was checked with a GLC quantitative standard (FAME Mix, Supelco, Inc., Bellefonte, PA, USA). Peaks corresponding to FAME were identified by a comparison of their retention times with those of a standard mixture (Qualmix Fish, Larodan, Malmo, Sweden). Peak areas were automatically integrated. Nonadecanoic FA (C19:0) (Sigma-Aldrich, St. Louis, MO, USA) was used as internal standard for quantitative purposes; for it, 100 μL (i.e., 40 μg C19:0) of a 0.4 mg·mL^−1^ solution in toluene was added to each sample before the methylation reaction with acetyl chloride. The content of each FA was calculated as g·100 g^−1^ of total FAs.

The results concerning the FA groups (STFA, MUFA, PUFA and total ω3 FA) and FA ratios (PUFA/STFA and ω3/ω6) were calculated on the basis of the quantification of the corresponding individual FAs mentioned in Table 2.

### 3.6. Statistical Analysis

The data (*n* = 5) obtained from moisture, lipid and FA determinations were subjected to a one-way ANOVA (*p* < 0.05) to investigate the differences resulting from canning procedure, packing medium and prior frozen storage (Statistica version 6.0, 2001; Statsoft Inc., Tulsa, OK, USA). A comparison of means was performed using a least-squares difference (LSD) method.

## 4. Conclusions

The present research checked the effect of the canning procedure and that of two common procedures carried out during canning (namely, packing medium addition and prior holding period). The FA composition of canned Atlantic mackerel showed a substantial effect of the canning procedure. A decrease (*p* < 0.05) in STFA levels, an increase (*p* < 0.05) in PUFA and total ω3 FA values and higher PUFA/STFA and ω3/ω6 ratio values were detected in canned fish. Concerning the effect of the packing medium, the great presence of C18:2ω6 in sunflower oil led to high PUFA and PUFA/STFA values and low ω3/ω6 ratios. However, the high presence of C18:1ω9 in both olive oils tested did not lead to remarkable increases (*p* > 0.05) in the presence of this FA in the corresponding canned mackerel muscle. Additionally, the contents of ω3 FAs, EPA and DHA did not provide remarkable differences as a result of the packing conditions used. Prior frozen storage did not have a substantial effect (*p* > 0.05) on FA group presence (STFA, MUFA, PUFA, total ω3) and FA ratio (PUFA/STFA and ω3/ω6) values.

The analysis of the FA composition of the resulting canned fish showed that the three factors maintained highly valuable FA contents, ω3/ω6 ratios being included in all cases in the 8.2–10.9 range and levels of EPA and DHA being included in 9.0–11.7 and 18.5–23.8 g·100 g^−1^ total FA ranges, respectively. According to the current nutritional recommendations, such scores can be considered highly valuable for human health and diet. On the basis of the great importance of canned seafood, further research ought to be carried out focused on the incidence of canning procedure, packing medium and prior holding time on other nutritional values (i.e., content on essential amino acids, vitamins, essential elements, etc.), as well as on the sensory and physical properties of canned fish related to quality.

## Figures and Tables

**Table 1:** Moisture and lipid content (g·kg^−1^ muscle) * in initial and canned mackerel muscle including different packing media **.

Constituent	Packing Medium	Mackerel Muscle		
		Initial	0-Canned	6-Canned
Moisture	Water	690.4 C (19.1)	652.6 Ba (13.0)	596.6 Aa (17.1)
	Brine	690.4 B (19.1)	674.1 Ba (21.6)	620.5 Aa (9.3)
	Sunflower oil	690.4 C (19.1)	659.3 Ba (8.0)	622.7 Aa (15.5)
	Refined olive oil	690.4 B (19.1)	643.3 Aa (12.2)	619.8 Aa (20.0)
	Virgin olive oil	690.4 C (19.1)	658.2 Ba (15.5)	619.0 Aa (17.8)
Lipids	Water	71.1 A (8.5)	115.2 Bb (14.9)	153.5 Cb (9.5)
	Brine	71.1 A (8.5)	96.6 Bb (14.6)	156.9 Cb (12.5)
	Sunflower oil	71.1 B (8.5)	51.1 Aa (6.9)	64.8 ABa (15.8)
	Refined olive oil	71.1 A (8.5)	60.1 Aa (5.5)	61.3 Aa (10.3)
	Virgin olive oil	71.1 A (8.5)	62.3 Aa (11.0)	65.8 Aa (13.6)

* Average values of five replicates (*n* = 5); standard deviations are indicated in brackets. In each row, average values followed by different capital letters (A,B,C) denote significant differences (*p* < 0.05) as a result of prior frozen storage and canning procedure. In each column, average values followed by different lowercase letters (a,b) denote significant differences (*p* < 0.05) as a result of packing medium. ** Abbreviations: 0-canned (canned fish without prior frozen storage) and 6-canned (canned fish with prior 6-month frozen storage).

**Table 2:** Fatty acid (FA) composition (g·100 g^−1^ total FAs) * of initial mackerel muscle and initial oils employed as packing media.

FA	Initial Oil-Packing Medium			Initial Mackerel Muscle
	Sunflower Oil	Refined Olive Oil	Virgin Olive Oil	
14:0	0.12 (0.01)	0.05 (0.00)	0.00 (0.00)	4.15 (0.45)
15:0	0.00 (0.00)	0.04 (0.00)	0.00 (0.00)	0.62 (0.08)
16:0	7.37 (0.01)	12.66 (0.04)	12.51 (0.00)	22.53 (0.85)
16:1ω7	0.15 (0.00)	0.79 (0.01)	0.92 (0.02)	5.16 (0.79)
17:0	0.06 (0.00)	0.09 (0.01)	0.12 (0.01)	1.06 (0.17)
18:0	4.21 (0.00)	3.24 (0.03)	3.33 (0.00)	5.44 (0.31)
18:1ω9	31.00 (0.01)	74.54 (0.01)	74.25 (0.02)	21.95 (3.69)
18:1ω7	0.79 (0.00)	2.03 (0.01)	2.24 (0.01)	4.89 (0.05)
18:2ω6	55.08 (0.02)	5.95 (0.01)	5.99 (0.02)	1.24 (0.15)
20:1ω9	0.20 (0.02)	0.28 (0.03)	0.26 (0.00)	2.67 (0.25)
20:2ω6	0.06 (0.00)	0.08 (0.01)	0.12 (0.04)	0.37 (0.04)
20:4ω6	0.86 (0.02)	0.16 (0.01)	0.15 (0.01)	1.00 (0.06)
22:1ω9	0.00 (0.00)	0.00 (0.00)	0.00 (0.00)	0.47 (0.05)
20:5ω3	0.10 (0.06)	0.05 (0.04)	0.04 (0.00)	8.48 (1.16)
22:4ω6	0.04 (0.00)	0.09 (0.01)	0.07 (0.00)	0.42 (0.08)
24:1ω9	0.00 (0.00)	0.00 (0.00)	0.00 (0.00)	0.70 (0.11)
22:5ω3	0.00 (0.00)	0.00 (0.00)	0.00 (0.00)	1.83 (0.36)
22:6ω3	0.00 (0.00)	0.00 (0.00)	0.00 (0.00)	17.03 (1.72)

* Average values of five independent determinations (*n* = 5); standard deviations are indicated in brackets.

**Table 3:** Values * for fatty acid (FA) groups (g·100 g^−1^ total FAs) and ratios in initial oils employed as packing media **.

FA Group/Ratio	Initial Oil-Packing Medium		
	Sunflower Oil	Refined Olive Oil	Virgin Olive Oil
STFA	11.76 aA (0.03)	16.06 bB (0.08)	15.96 bB (0.01)
MUFA	32.14 aB (0.02)	77.63 bC (0.04)	77.67 bC (0.01)
PUFA	56.10 bC (0.01)	6.31 aA (0.04)	6.37 aA (0.01)
PUFA/STFA	4.77 b (0.01)	0.39 a (0.00)	0.40 a (0.00)
Total ω3	0.10 b (0.06)	0.03 a (0.01)	0.04 a (0.00)
Total ω6	56.00 b (0.06)	6.28 a (0.00)	6.33 a (0.01)
ω3/ω6 ratio	0.00 a (0.00)	0.00 a (0.00)	0.01 a (0.00)

* Average values of five independent determinations (*n* = 5); standard deviations are indicated in brackets. Abbreviations: STFA (saturated FAs), MUFA (monounsaturated FAs) and PUFA (polyunsaturated FAs). ** In each row, values followed by different lowercase letters (a,b) indicate significant differences (*p* < 0.05) among the three initial oils. For each initial oil, different capital letters (A,B,C) denote significant differences (*p* < 0.05) among FA groups (STFA, MUFA and PUFA).

**Table 4:** Determination * of fatty acid (FA) group values (g·100 g^−1^ FAs) in initial and canned mackerel muscle including different packing media **.

FA Group	Packing Medium	Mackerel Muscle		
		Initial	0-Canned	6-Canned
STFA	Water	33.79 B (0.34)	28.23 Aa (0.56)	29.89 Aa (2.72)
	Brine	33.79 B (0.34)	28.50 Aa (0.56)	28.32 Aa (0.91)
	Sunflower oil	33.79 B (0.34)	27.79 Aa (0.34)	28.94 Aa (0.78)
	Refined olive oil	33.79 B (0.34)	27.57 Aa (0.47)	28.43 Aa (1.35)
	Virgin olive oil	33.79 B (0.34)	29.30 Aa (2.35)	28.62 Aa (1.06)
MUFA	Water	35.85 A (2.82)	36.24 Ab (0.87)	32.59 Aab (3.77)
	Brine	35.85 A (2.82)	35.31 Aab (3.89)	35.96 Ab (1.13)
	Sunflower oil	35.85 B (2.82)	31.33 Aa (1.70)	31.47 Aa (1.36)
	Refined olive oil	35.85 A (2.82)	37.33 Ab (1.71)	34.31 Aab (1.72)
	Virgin olive oil	35.85 A (2.82)	35.36 Ab (0.56)	34.30 Ab (0.70)
PUFA	Water	30.36 A (2.04)	35.52 Ba (0.46)	37.51 Bab (2.26)
	Brine	30.36 A (2.04)	36.19 Bab (2.76)	35.72 Ba (1.30)
	Sunflower oil	30.36 A (2.04)	40.88 Bb (1.73)	39.59 Bb (2.06)
	Refined olive oil	30.36 A (2.04)	35.09 Ba (1.58)	37.26 Bab (1.46)
	Virgin olive oil	30.36 A (2.04)	35.32 Bab (2.32)	37.08 Bab (0.54)

* Average values of five replicates (*n* = 5); standard deviations are indicated in brackets. In each row, average values followed by different capital letters (A,B) denote significant differences (*p* < 0.05) as a result of prior frozen storage and canning procedure. For each FA group and in each column, average values followed by different lowercase letters (a,b) denote significant differences (*p* < 0.05) as a result of packing medium. ** Abbreviations: STFA (saturated FAs), MUFA (monounsaturated FAs), PUFA (polyunsaturated FAs), 0-canned (canned fish without prior frozen storage) and 6-canned (canned fish with prior 6-month frozen storage).

**Table 5:** Determination (g·100 g^−1^ total FAs) of EPA, DHA and total ω3 fatty acid values * in initial and canned mackerel muscle including different packing media **.

FA Group	Packing Medium	Mackerel Muscle		
		Initial	0-Canned	6-Canned
Total ω3	Water	27.33 A (3.04)	32.10 Ba (0.40)	34.33 Ba (3.85)
	Brine	27.33 A (3.04)	33.16 Aab (3.68)	32.67 Aa (1.19)
	Sunflower oil	27.33 A (3.04)	36.65 Bb (1.76)	33.45 ABa (3.09)
	Refined olive oil	27.33 A (3.04)	32.09 Ba (1.57)	33.82 Ba (1.54)
	Virgin olive oil	27.33 A (3.04)	32.13 Ba (1.38)	33.68 Ba (0.60)
EPA	Water	8.48 A (1.16)	11.32 Bb (0.67)	11.73 Ba (1.73)
	Brine	8.48 A (1.16)	11.32 Bb (0.81)	11.14 Ba (1.06)
	Sunflower oil	8.48 A (1.16)	10.61 Aa (1.19)	11.58 Aa (2.44)
	Refined olive oil	8.48 A (1.16)	9.57 Aab (2.10)	10.56 Aa (1.32)
	Virgin olive oil	8.48 A (1.16)	8.96 Aa (1.73)	10.68 Aa (1.50)
DHA	Water	17.03 A (1.72)	18.54 Aa (0.48)	19.92 Aa (2.96)
	Brine	17.03 A (1.72)	19.31 Aab (3.88)	18.96 Aa (1.90)
	Sunflower oil	17.03 A (1.72)	23.79 ABb (2.59)	19.30 Ba (1.86)
	Refined olive oil	17.03 A (1.72)	20.34 Bb (1.09)	20.98 Ba (1.11)
	Virgin olive oil	17.03 A (1.72)	21.15 Bb (1.57)	20.75 Ba (1.41)

* Average values of five replicates (*n* = 5); standard deviations are indicated in brackets. In each row, average values followed by different capital letters (A,B) denote significant differences (*p* < 0.05) as a result of prior frozen storage and canning procedure. In each column and for each ratio, average values followed by different lowercase letters (a,b) denote significant differences (*p* < 0.05) as a result of packing medium. ** Abbreviations: EPA (eicosapentaenoic acid) and DHA (docosahexaenoic acid).

## Data Availability

Not applicable.
